# Supplementary material for: Chromothripsis during telomere crisis is independent of NHEJ, and consistent with a replicative origin
Source: Genome Res. 2019 May;29(5):737–49. doi: 10.1101/gr.240705.118 (PMC6499312; doi:10.1101/gr.240705.118)
Supplement: Supplemental Material [file supp_gr.240705.118_Supplemental_file_1.zip › contigs/annotated_contigs/DB84/contig.2.DB84_length_876_mean_cov_8.56164383562.docx]

**DB84_length_876_mean_cov_8.56164383562**

```
GGAATGGAAAGGAAGAAGTAAGGAGAAAATGGTTGTTAAATAGTGGGTGAGAGTCTATACATTAAAAGGAGAGCGTGCATGCTCTAATG
 >chr1:68732460-68732772 - E=6e-171
TACTTTATAATAAATGTGGTCTATTTGATTAATGCTGCCAGCTCAAATTTGGGTCTTCTGGAAAAAAAAATGATGAAACCATCTCCGGA

GACATTTTGTATTCAGTTCATTTAATATTTATACTGAGTTTCTTAAGTCAGCATCCTCAAGGAGACCAAAAATGATGGTTGGAATATGG

AGAGTAATGCTCAGGAGGATGGGGTAAGAGCACTCCCCACTCAC|TATACTATACAAAATAA|CTATACTAGTTATTTTGAAGTCTTTG
                                                              >chr1:68836038-68836586 -
CTCCACCAAATCTAACATCTGTCCCATCAGAAACAGTTTCCATTGTTTTTCTTTCTGTGTGTGGTCACACTTTCCTGGTTTTTTGCATG
 E=9e-312
TCATTTCTTTAACTGGACATTTTAGATAATATATTGTAGTAACTCTGGATTCGTCTCCTCTCCAGGGTTAGTTGCTGTTGCTACTTTTT

TGATTTGCTGAATGACTTGCCTGGATAAATTCTGTAGAATCTATCTCACAACAGTGTGCAGCTCCTGTTGTTTCTGTTCAGGTTCCTTT

TTAAAATTTATTTTTGTTTAATTTTTAAATCTGGCTTCCCATGAGTCACTCCTGGATTAGCATAGCTTAATGATCAGCCAAAAATTGAT

CAGAGGTTGCACATAAACGTATTGAGCCAGTAAGCCTTCTGCTCTTTGCCAACAGATCTGTGTGTTTAAGGAATGTAATCAAGGCTGGG

CATGGTGGCTCACACCCTGTAATCCCAGCACTTTAGGAGGCCAAAGCAAGAGGATCACTTGAGACTAGCAGTTTAAG
```
